# ASO Author Reflections: Impact of Social Vulnerability and Allostatic Load on Postoperative Outcomes

**DOI:** 10.1245/s10434-025-18274-w

**Published:** 2025-08-27

**Authors:** Mujtaba Khalil, Timothy M. Pawlik

**Affiliations:** 1 Department of Surgery, School of Medicine and Public Health, University of Wisconsin, Madison, WI USA; 2 Department of Surgery, The Ohio State University Wexner Medical Center and James Comprehensive Cancer Center , Columbus, OH USA

## Past

Hepatopancreatobiliary (HPB) cancers remain among the most complex malignancies to treat surgically, with high postoperative morbidity and mortality.^1^ For patients living in socially vulnerable neighborhoods, the challenges extend beyond the operating room.^2,3^ Chronic exposure to socioeconomic stressors such as financial hardship, limited healthcare access, and unstable living conditions can alter the body’s regulatory systems, a process captured by the concept of allostatic load (AL).^4^ AL reflects cumulative physiologic wear and tear across cardiovascular, metabolic, renal, and immune systems.^4^ While prior work has linked AL to poor outcomes in chronic medical conditions, its influence on patients undergoing high-risk HPB surgery has been ill-defined.^4,5^

## Present

Among 34,253 individuals, the mean patient age was 71 years (interquartile range 63–78), approximately half of the patients were male (*n* = 18,045, 52.7%), and 29,246 (85.4%) patients had a high Charlson Comorbidity Index score (CCI >2). The most common cancer site was the pancreas (*n* = 21,402, 62.5%), followed by the liver (*n* = 8451, 24.7%) and biliary tract (*n* = 4400, 12.8%). Overall, 13.8% (*n* = 4717) of patients had high AL. On multivariable analysis, the risk of allostasis increased stepwise with higher social vulnerability (reference: low; medium: odds ratio [OR] 1.11, 95% confidence interval [CI] 1.04–1.19; high: OR 1.17, 95% CI 1.11–1.17). Moreover, high AL was associated with a 44% increased risk of Clavien–Dindo grade IV complications (OR 1.44, 95% CI 1.36–1.54) and an 85% increased risk of failure to rescue (OR 1.85, 95% CI 1.60–2.13). In addition, the risk of 30-day mortality was approximately twofold higher with elevated AL (OR 1.92, 95% CI 1.70–2.19).

## Future

The current study highlights the need to recognize and address biologic consequences of long-term social disadvantage in patients with cancer.^5^ AL, derived from routinely collected clinical data, could be integrated into preoperative workflows to identify patients who may benefit from targeted interventions ranging from prehabilitation and metabolic optimization to enhanced perioperative surveillance and tailored discharge planning.^4^ Future studies should explore whether modifying AL before surgery can improve recovery and survival, and how combining patient-level physiologic data with hospital-level quality measures might further reduce disparities in HPB cancer care.^4,5^
